# A Movement Has Been Started

**Published:** 1899-10

**Authors:** 


A MOVEMENT has been started among Philadelphia women, the aim of which is to wage war on disease and its prevention by practical rules of hygiene. A meeting has been held, organization effected and a charter applied for. The organization will be known as the "Woman's Sanitary League of Pennsylvania."

Its purpose is the advancement of sanitary science, the promotion of public health and good government, and the forming of country and local auxiliaries to extend the beneficent work. It is stated that the action of the St. Louis Board of Health in requiring the disinfection of all such materials as second-hand books and clothing is the precedent with which the committee presents its plea to the Philadelphia authorities. The subject of schools and school children, expectoration and the summer diseases of childhood will all come in for their share of consideration.—*Journal American Medical Association.*

